# Higher iron stores and the *HFE* 187C>G variant delay onset of peripheral neuropathy during combination antiretroviral therapy

**DOI:** 10.1371/journal.pone.0239758

**Published:** 2020-10-15

**Authors:** Asha R. Kallianpur, Wanqing Wen, Angelika L. Erwin, David B. Clifford, Todd Hulgan, Gregory K. Robbins

**Affiliations:** 1 Genomic Medicine Institute, Cleveland Clinic/Lerner Research Institute, Cleveland, Ohio, United States of America; 2 Department of Molecular Medicine, Cleveland Clinic Lerner College of Medicine of Case Western Reserve University, Cleveland, Ohio, United States of America; 3 Department of Population and Quantitative Health Sciences, Case Western Reserve University School of Medicine, Cleveland, Ohio, United States of America; 4 Division of Epidemiology, Department of Medicine, Vanderbilt University Medical Center, Nashville, Tennessee, United States of America; 5 Division of Infectious Diseases, Departments of Medicine and Neurology, Washington University School of Medicine, Saint Louis, Missouri, United States of America; 6 Division of Infectious Diseases, Department of Medicine, Vanderbilt University Medical Center, Nashville, Tennessee, United States of America; 7 Division of Infectious Diseases, Department of Medicine, Massachusetts General Hospital, Boston, Massachusetts, United States of America; University of Pennsylvania, UNITED STATES

## Abstract

**Objective:**

People with HIV (PWH) continue to experience sensory neuropathy and neuropathic pain in the combination antiretroviral therapy (cART) era for unclear reasons. This study evaluated the role of iron in a previously reported association of iron-loading hemochromatosis (*HFE*) gene variants with reduced risk of neuropathy in PWH who received more neurotoxic cART, since an iron-related mechanism also might be relevant to neuropathic symptoms in PWH living in low-resource settings today.

**Design:**

This time-to-event analysis addressed the impact of systemic iron levels on the rapidity of neuropathy onset in PWH who initiated cART.

**Methods:**

Soluble transferrin receptor (sTFR), the sTFR-ferritin index of iron stores, and high-sensitivity C-reactive protein (hsCRP) levels were determined in stored baseline sera from participants of known *HFE* genotype from AIDS Clinical Trials Group (ACTG) Study 384, a multicenter randomized clinical trial that evaluated cART strategies. Associations with incident neuropathy were evaluated in proportional-hazards, time-to-event regression models, adjusting for potential confounders.

**Results:**

Of 151 eligible participants with stored serum who were included in the original genetic study, 43 had cART-associated neuropathy; 108 had sufficient serum for analysis, including 30 neuropathy cases. Carriers of *HFE* variants had higher systemic iron (lower sTFR and sTFR-ferritin index) and lower hsCRP levels than non-carriers (all *p*<0.05). Higher sTFR or iron stores, the *HFE* 187C>G variant, and lower baseline hsCRP were associated with significantly delayed neuropathy in self-reported whites (n = 28; all *p*-values<0.05), independent of age, CD4+ T-cell count, plasma HIV RNA, and cART regimen.

**Conclusions:**

Higher iron stores, the *HFE* 187C>G variant, and lower hsCRP predicted delayed onset of neuropathy among self-reported white individuals initating cART. These findings require confirmation but may have implications for cART in HIV+ populations in areas with high endemic iron deficiency, especially those PWH in whom older, more neurotoxic antiretroviral drugs are occasionally still used.

## Introduction

Combination antiretroviral therapy (cART) has transformed clinical outcomes in HIV infection, and the incidence of peripheral sensory neuropathy has clearly declined as older, dideoxynucleoside reverse-transcriptase inhibitor drugs (dNRTIs) have been largely replaced by less neurotoxic drugs. HIV sensory neuropathy remains problematic, however, among people living with HIV (PWH) globally, particularly in developing countries and resource-limited settings, where dNRTIs are still used [1, 2]. HIV sensory neuropathy is a painful and debilitating complication of HIV infection and its treatment with older dNRTIs, such as stavudine, and neuropathic pain occurs in up to 40% of all people with HIV (PWH), including those on contemporary cART regimens [3, 4]. Predisposing factors for neuropathy include older age, greater height, prior neurotoxic exposures, advanced HIV disease, statin and fibrate use, diabetes, hypertriglyceridemia, and poor nutrition [1, 2, 5]. Host genetic factors, including variant cytokine genotypes and specific mitochondrial DNA haplogroups, have also been associated with susceptibility to sensory neuropathy in HIV-seropositive (HIV+) persons [3, 4, 6, 7]. HIV-related neuropathy and neuropathic pain are continuing challenges in HIV clinics worldwide, especially in settings where second-line cART is in use [8–10]. Stavudine has still been used in some low-resource settings, due to its lower cost and availability, though its use has declined [11–17]. It should also be noted that some studies have linked protease inhibitors such as indinavir to increased risk of HIV sensory neuropathy [9, 18, 19]. Finally, while rates of incident, symptomatic neuropathy in PWH may be significantly lower when the CD4+ T-cell count is >250 and newer antiretroviral drugs are initiated, these findings may not apply to areas where nutritional deficiencies, which contribute to neuropathy risk, are endemic. A recent study conducted in South Africa, for example, indicated that the incidence of HIV neuropathy remains significant within the first 6 months of starting contemporary cART in the post-stavudine era (approximately 17% overall, 8% of cases being symptomatic) [20].

Mitochondrial toxicity by dNRTIs has long been implicated in the etiology of HIV-associated neuropathy [21–23]. Neurons are dependent on mitochondrial function, which in turn relies on a steady and carefully regulated iron supply [24, 25]. Cellular iron transport is altered in HIV infection by a combination of inflammation-related, and HIV *Nef*-mediated, iron sequestration within the monocyte-macrophage compartment, which limits iron release from macrophages to metabolically active cells and possibly promotes both HIV replication and antiretroviral neurotoxicity [26]. The gene *HFE*, which when mutated may cause the systemic iron overload disorder *hereditary hemochromatosis*, influences HIV-mediated iron dysregulation: the *HFE* gene product normally competes with transferrin for binding to the transferrin receptor (TFR), reducing its affinity for iron-bound transferrin and lowering macrophage iron content, but HIV *Nef* downregulates the expression of *wildtype* HFE protein on the cell surface, counteracting these effects and leading to elevated systemic indices of iron (*e*.*g*., serum ferritin and/or transferrin saturation) [27]. HFE also plays a role in regulating iron release via mechanisms that involve the hepatic antimicrobial peptide hormone, hepcidin, but these functions are not completely understood [28–30]. In homozygotes for the iron-loading hemochromatosis (*HFE*) gene variant 845G>A (C282Y), macrophages and monocytes cannot sequester iron; nor do they exhibit the previously described *Nef*-mediated changes in iron transport [26]. The more prevalent 187C>G (H63D) *HFE* variant appears to have synergistic effects on cellular iron transport when present together with *HFE* 845G>A, but its independent impact on iron metabolism is less clear [31–33]. Both *HFE* 845G>A and 187C>G are much more prevalent among individuals of northern European ancestry. We previously reported that heterozygosity for *HFE* 845G>A was independently associated with reduced risk of dNRTI-associated neuropathy among AIDS Clinical Trials Group (ACTG) Study 384 participants [34]. A non-significant but similarly protective effect of *HFE* 187C>G was found among self-reported whites in that study (adjusted odds ratio 0.50, *p* = 0.078).

Since *HFE* is a major histocompatibility complex (MHC) Class I-linked gene, neuroprotective effects of this variant might be due to down-modulation of inflammation by other MHC Class I-linked genes rather than to *HFE*-mediated effects on iron transport or iron levels. This study specifically addressed the impact of circulating iron indices and estimated systemic iron stores on HIV-associated peripheral sensory neuropathy, by evaluating both measures of inflammation and iron status in relation to the rapidity of onset of neuropathy in ACTG Study 384 participants after starting cART. We found that measures indicating higher systemic iron at the cellular level or higher systemic iron stores, as well as the minor *HFE* gene variant were associated with delayed onset of neuropathy among PWH on cART.

## Patients and methods

### Study participants

ACTG Study 384 was a multicenter clinical trial designed to assess initial treatment strategies in HIV+ adults and has been described in detail previously [35, 36]. Briefly, treatment-naïve HIV-positive participants were randomized to receive different four-drug or sequential three-drug treatment regimens. The present study is a further analysis of baseline sera collected at the time of enrollment from ACTG Study 384 participants who were included in our previous genetic study [34]. Ascertainment of neuropathy cases and controls in that study has been described elsewhere [34]; briefly, neuropathy cases were PWH who developed clinical signs and/or symptoms of peripheral nerve toxicity of grade 1 severity or higher, as defined by the publicly available Division of AIDS (DAIDS) table for grading the severity of adult and pediatric adverse events, version 1.0. Time of onset of neuropathy was determined as the time elapsed between entry onto ACTG Study 384 (when cART was initiated) and the date of onset of self-reported clinical symptoms and/or elicited signs of neuropathy (whichever occurred earlier). Clinical symptoms and signs of neuropathy were ascertained at visits 16 weeks apart for up to 2.3 years on the study, which had a median follow-up of 28 months and median duration of treatment with study drugs of 27 months; mean self-reported adherence was reportedly >97% [35]. Written informed consent for collection of specimens and data was obtained from all ACTG Study 384 participants, and this analysis was approved by the Vanderbilt University Medical Center Institutional Review Board. All study protocols were conducted in accord with the Declaration of Helsinki.

### Serum assays

Serum samples, collected at entry and stored at -80°C, were obtained from the ACTG Biospecimen Repository for measurement of soluble transferrin receptor (sTFR), and serum ferritin. sTFR and serum ferritin were measured by chemiluminescence and ELISA immunoassay, respectively. *HFE* genotypes at the G845A and C187G loci were determined as previously described [34]. The sTFR)-ferritin index was calculated using the formula: sTFR-ferritin index = (sTFR)/log_10_[serum ferritin] [37, 38]. The sTFR is a sensitive indicator of tissue iron demand at the tissue or cellular level, and it is unaffected by infections and significantly less affected by inflammation than other iron biomarkers [39]. The sTFR-ferritin index is a more robust noninvasive index of systemic iron stores than serum ferritin, as it adjusts for the effects of inflammation. Both indices (sTFR and the sTFR-ferritin index) are inversely proportional to total body iron stores: higher levels of sTFR or the sTFR-ferritin index reflect lower iron stores. Inflammation was assessed by measuring serum high-sensitivity C-reactive protein (hsCRP) levels by latex immunoturbidimetry assay. The hsCRP, serum ferritin and sTFR assays were all performed at ARUP Laboratories, Inc. (Salt Lake City, UT): test numbers 0070283 (sTFR), 0070065 (SF), and 0050182 (hsCRP).

### Statistical analyses

Demographic and clinical characteristics of study participants were compared using Fisher’s exact test for categorical variables. Determination of racial ancestry was based on self-report. The Shapiro-Francia test was used to test normality of variable distributions. The Student’s *t*-test and either the Wilcoxon rank sum test or the equality of medians test were used to compare normally and non-normally distributed continuous variables, respectively. Cox proportional-hazards regression models were used to estimate hazard ratios (HRs) for time to event (onset of neuropathy), adjusting for potential confounders. Statistical analyses were performed using STATA software, version 12.0 (College Station, TX). All tests were two-tailed, and *p*-values<0.05 were considered statistically significant.

## Results

Serum samples collected prior to initiating cART [didanosine (ddI) and stavudine (d4T) or zidovudine (ZDV) plus lamivudine (3TC), with nelfinavir (NFV), efavirenz (EFV) or both] were obtained from 151 ACTG Study 384 participants who had previously provided DNA (and a subset of the prior genetic study participants) and who also had available serum. Of these 151, including 44 with neuropathy from our prior analysis, sufficient serum for iron-related assays and hsCRP quantification was available in 110, of whom 108 (98.2%) had *HFE* genotype data (including 30 of the neuropathy cases). In this subset of 108 PWH, 33 carriers of at least one *HFE* variant allele were identified, including 10 who were heterozygous for the *HFE* 845G>A variant only, 21 who were heterozygous for 187C>G only, and 2 compound heterozygotes (with both variant alleles). The remaining individuals had only ancestral (*wildtype*) *HFE* alleles.

Baseline characteristics of the 43 neuropathy cases with *HFE* genotype data are shown in Table 1. Mean age of the sample was 38.8 (SD 8.5) years, 7 (16.3%) were women, 15 (34.9%) were non-whites or “other” race/ethnicity by self-report, median CD4+ T-cell count was 265 [interquartile range (IQR) 65, 429], median HIV RNA concentration (viral load) in plasma was 5.1 (IQR 4.6, 5.6) log_10_(copies/mL), and 32 (74%) had been randomized to the ddI/d4T-containing treatment arm at enrollment. The median time to onset of neuropathy in all 43 cases was 259 days (IQR, 148–425 days). *HFE* genotype was not significantly associated with time to onset of neuropathy (dichotomized), although a higher proportion of individuals with onset of neuropathy at or greater than the median had *HFE* variant alleles. Significantly higher CD4+ T-cell counts and a lower likelihood of randomization to the ddI/d4T arm were observed among individuals with later onset of neuropathy (≥259 days). Age, sex, race/ethnicity, and viral load were not significantly related to time to onset of neuropathy.

**Table 1:** Baseline characteristics of 43 ACTG Study 384 participants with *HFE* genotype data who developed peripheral neuropathy, stratified by time to onset.

	Onset < median (n = 21)	Onset ≥ median (n = 22)	*p*-value^a^
Age (years) *mean (SD)*	39.5 (9.5)	38.1 (8.5)	0.61
Self-reported race/ethnicity n (% non-white or “other”)	8 (38.1)	7 (31.8)	0.47
Sex, n (% women)	3 (14.3)	4 (18.2)	0.73
CD4^+^ T-cell count (cells/mm^3^), *median (IQR)*	133.2 (33, 370.5)	337.5 (161, 430)	0.02
Plasma HIV RNA Log_10_(copies/mL), *median (IQR)*	5.1 (4.6, 5.6)	4.9 (4.4, 5.2)	0.23
Randomization to ddI+d4T, n (%)	19 (86.4)	13 (59.1)	0.04
≥1 *HFE* variant, n (%)	5 (23.8)	10 (45.5)	0.14
845GA	1 (4.8)	3 (13.6)	
187CG or GG	4 (19.0)	8 (36.4)^b^	

Median onset of peripheral neuropathy overall was 259 days (398 days for carriers of *HFE* variant alleles, and 252 days for those with ancestral *HFE* alleles). More neuropathy cases than controls were randomized to the didanosine (ddI) arm and CD4+ T-cell counts were higher in individuals with slower onset. The proportion of participants with ≥1 *HFE* gene variant was nonsignificantly higher in the group with delayed-onset neuropathy.

^a^Equality of medians test was used for comparison of variables that were not normally distributed; Student’s *t*-test for normally distributed variables; Chi-squared tests for between-group comparison of categorical variables. Two-tailed *p*-values <0.05 are considered significant.

^b^One person was a compound heterozygote with both 845A and 187G alleles and is therefore included in both genotype groups.

*Abbreviations*: *SD*, standard deviation; *ddI*, didanosine; *IQR*, interquartile range.

Carriers of at least one *HFE* variant allele had higher iron stores (reflected by lower sTFR levels and sTFR-ferritin index) and lower levels of hsCRP than non-carriers (Table 2a; all *p*<0.05). Time to onset of neuropathy was non-significantly longer in individuals with *HFE* variants (median 398 days, IQR 216–531 *vs*. 252 days, IQR 148–345; *p* = 0.15). As shown in Table 2b, 17 neuropathy cases (30.9%) occurred in the group with a sTFR -ferritin index below the median (higher iron stores), compared to 13 cases (23.6%) in the group with sTFR -ferritin indices at or above the median (lower iron stores), a similarly non-significant difference (*p* = 0.39, Chi-squared test). However, median hsCRP levels tended to be higher (1.2 mg/L, IQR 0.6–2.4) in those with a higher sTFR-ferritin index (lower iron stores) than in individuals with a lower iron index (0.87 mg/L, IQR 0.5–2.2; *p* = 0.056). Time to onset of neuropathy in these 30 individuals, 28 of whom were self-reported white, was also non-significantly shorter in the subset with a higher sTFR -ferritin index (median 147 days, IQR 94–261 *vs*. 251 days (IQR 166–327, respectively; *p =* 0.25) in unadjusted analyses. Neither the sTFR-ferritin index nor sTFR levels were significantly correlated with hsCRP levels at baseline.

**Table 2:** Unadjusted analyses of baseline iron status, inflammation, and rapidity of onset of neuropathy in ACTG Study 384 participants with *HFE* genotype and/or serum iron indices.

**(a)**	**≥1 *HFE* variant (n = 33) *median (IQR)***	**0 *HFE* variants (n = 75) *median (IQR)***	***p*-value**^b^
sTFR (mg/L), *median (IQR)*	3.1 (2.7–3.7)	3.9 (3.3–4.7)	**<**0.01
sTFR-ferritin index^a^, *median (IQR)*	1.3 (0.0–1.8)	1.4 (1.1–1.8)	<0.01
hsCRP (mg/L), *median (IQR)*	0.78 (0.45–1.4)	1.2 (0.6–2.6)	0.03
Time to onset of neuropathy in cases, days *median (IQR)*^c^	398 (216–531)	252 (148–345)	0.15
**(b)**	**sTFR-ferritin < median**^a^ **(n = 55)**	**sTFR-ferritin index ≥ median**^a^ **(n = 55)**	***p-value***^b^
Neuropathy, n (%)	17 (30.9)	13 (23.6)	0.39
hsCRP (mg/L), *median (IQR)*	0.87 (0.5–2.2)	1.2 (0.6–2.4)	0.06
Time to onset in cases, days, *median (IQR)*^c^	251 (166–327)	147 (94–261)	0.25

Individuals with at least one *HFE* variant had higher iron stores (lower sTFR and sTFR-ferritin index) and less inflammation than individuals who did not carry these variants. Inflammatory biomarker levels tended to be higher in individuals with a higher sTFR-ferritin index (lower iron stores). The proportion of neuropathy cases was non-significantly lower in people with lower iron stores (higher sTFR-ferritin index), and time to onset of neuropathy was more rapid in this subset (also not statistically significant).

^a^The sTFR -ferritin index was calculated using the modified Cook’s formula: sTFR/ log_10_(serum ferritin).

^b^*p*-values are from the Student’s *t*-test (normally distributed continuous variables), the Wilcoxon Rank Sum test (skewed continuous variables), or the Chi-square test (count data); *p*-values <0.05 are considered statistically significant.

^c^Neuropathy cases only (N = 30 with *HFE* genotype and serum iron data).

*Abbreviations*: sTFR, soluble transferrin receptor (higher values indicate lower tissue or cellular iron); hsCRP, high-sensitivity C-reactive protein; IQR, interquartile range.

Results of analyses adjusted for age, CD4+ T-cell count, plasma HIV RNA, and randomization (yes/no) to the ddI/d4T-containing arm are presented for all 43 neuropathy cases with genotype data, and for 28 self-reported whites with neuropathy, *HFE* and serum iron indices, in Table 3. These results revealed a significantly slower onset of neuropathy associated with *HFE* 187C>G as compared to *HFE* 187C [adjusted hazard ratio (HR) 0.47, *p* = 0.041]. Since the *HFE* variants previously associated with reduced neuropathy are uncommon among PWH of non-European ancestry, predictors of time to diagnosis of neuropathy were also evaluated in the subset of 28 self-reported white cases in separate multivariable, time-to-event Cox regression models [40]. Presence of at least one *HFE* 187C>G allele predicted slower onset of neuropathy (HR 0.41, 95% CI 0.17–1.02; *p* = 0.054); there were too few *HFE* 845G>A carriers to evaluate this subset separately. Higher hsCRP was associated with more rapid onset of neuropathy [HR 1.65 per unit rise in hsCRP (95% CI 1.0–2.7; p = 0.040)]. Lower iron stores (higher sTFR or sTFR-ferritin index) also predicted significantly more rapid development of neuropathy during cART, independent of other factors [HR 2.8 per unit rise (95% CI 1.5–5.5), *p* = 0.002 for sTFR and 29.5 per unit rise (95% CI 3.2, 270.1), *p* = 0.003 for sTFR-ferritin index]. Based on sTFR and serum ferritin measurements, absolute iron deficiency and iron overload were not observed in this study sample. Overall results of multivariable-adjusted analyses of time to onset of neuropathy were not significantly altered for either sTFR or the sTFR-ferritin index by including either sex or hsCRP levels as covariates in regression models (Table 3).

**Table 3:** Multivariable-adjusted hazard ratios (HRs) for time to onset of neuropathy among neuropathy cases.

Variable	Adjusted HR^a^ (95% CI), *median (IQR)*	*p*-value^b^
*All cases with HFE genotype data (N = 43)*		
*HFE* 845GA *vs*. GG	0.96 (0.33–2.81)	0.94
*HFE* 187CG or GG *vs*. CC	0.47 (0.23–0.97)	0.04
*Self-reported white cases with HFE genotype and serum iron data (N = 28)*		
*HFE* 845GA *vs*. GG	1.08 (0.35–3.33)	0.90
*HFE* 187CG or GG *vs*. CC	0.41 (0.17–1.02)	0.05^c^
sTFR (mg/L)	2.81 (1.45–5.45)	<0.01^d^
sTFR-ferritin index	29.5 (3.23–270.1)	<0.01^d^
hsCRP (mg/L)	1.65 (1.02–2.67)	0.04

Among all neuropathy cases, *HFE* 187CG or GG genotype was associated with a significantly longer time to neuropathy onset than the ancestral CC genotype. Higher levels of the inflammatory biomarker hsCRP were associated with more rapid onset. In self-reported whites with adequate serum for analysis, *HFE* 187C>G was still associated with delayed neuropathy onset; higher sTFR and sTFR -ferritin index, both reflecting lower iron stores, and higher hsCRP levels, were all associated with more rapid onset.

^a^Hazard ratio>1 indicates more rapid onset of neuropathy, and HR<1 indicates slower onset. Models were adjusted for age, plasma HIV RNA, CD4+ T-cell count, and randomization to ddI treatment.

^b^Each variable was evaluated independently, each row representing a separate multivariable model, adjusted for the above-mentioned baseline factors.

^c^*p*-value = 0.054.

^d^*p*-values remained <0.01 for sTFR and <0.05 for the sTFR-ferritin index, if sex or hsCRP were included in the regression model.

*Abbreviations*: *HR*, hazard ratio; *sTFR*, soluble transferrin receptor (low concentration indicates high iron stores; *hsCRP*, high-sensitivity C-reactive protein.

## Discussion

Persistence of HIV sensory peripheral neuropathy in the current cART era is consistent with an inflammatory or inflammatory-metabolic pathogenesis [41]. We previously reported a significantly reduced risk of dNRTI-related neuropathy among PWH who were carriers of common iron-loading *HFE* gene variants 187C>G and 845G>A in ACTG Study 384 [34]. Potential mechanisms for this association include a protective effect of higher iron levels (a known consequence of *HFE* variants), non-*HFE* MHC Class I haplotype-mediated downregulation of inflammation causing reduced development of neuropathy, or both. In this analysis, lower cellular iron (reflected by higher sTFR values) and lower systemic iron stores (higher sTFR-ferritin index) at the time of initiating cART, and the *HFE* 187C>G variant, were independently associated with more rapid onset of peripheral neuropathy in self-reported whites, and findings confirmed that PWH who had *HFE* variants also had higher iron stores. More importantly, our results suggest that higher cellular and systemic iron levels, not simply reduced inflammation related to *HFE* variants, may explain the reduced risk of cART-associated neuropathy that was observed with these variants in PWH. In addition, the current analysis included ACTG Study 384 participants exposed to cART that did not include the more neurotoxic dNRTIs; therefore, our findings may also advance understanding of HIV sensory neuropathy in PWH on more modern regimems, as well as PWH at risk for this condition in low-resource settings who receive older antiretroviral drugs [1, 20].

Despite the small sample size, lower systemic iron stores (higher sTFR-ferritin index) independently predicted more rapid onset of neuropathy, with a potentially clinically meaningful delay in onset of 3 to 6 months. This observation suggests that lower iron stores at the time of initiating cART may increase the likelihood of neurotoxicity, and that avoiding iron stores in the low-normal or deficient range may be beneficial. Assessing sTFR levels and/or systemic iron status before initiating cART may be a useful strategy, especially in areas of endemic iron deficiency, in women during their childbearing years, and in children [8, 42, 43]. Poorer HIV-related outcomes have been reported in individuals with anemia with or without iron deficiency, as well as iron excess, but participants in some of these studies did not receive antiretroviral therapy and/or had other significant comorbidities [44–49]. Higher iron levels in PWH in care in whom cART is initiated may reduce mitochondrial toxicity to peripheral nerves, which occurs as a result of cART- and/or HIV-mediated changes in iron homeostasis, and the direct effects of HIV proteins on mitochondrial function [4, 50]. The same changes may also contribute to chronic neuropathic pain in PWH, regardless of the type of cART. We previously reported associations between iron-regulatory or iron-transport genes including *HFE*, and occurrence of painful neuropathy in PWH on cART [51]. In that study, individuals with neuropathy of any severity were significantly more likely to be taking protease inhibitors, and persons who reported neuropathic pain tended to be female and were substantially less likely to be cART-naïve. Investigation of the impact of iron status on clinical outcomes, including non-HIV infections, in larger numbers of PWH (including women) and more diverse cohorts of individuals starting cART, are needed before recommendations regarding iron testing or iron supplementation during cART can be made [49].

Higher cellular iron and body iron stores, and lower pre-existing inflammation, appear to delay development of HIV-associated neuropathy, and these measures are linked via the hepcidin-ferroportin pathway. The iron-regulatory hormone hepcidin, produced in the liver in response to pro-inflammatory stimuli and increased iron levels, prevents gut iron absorption and reduces macrophage expression of the iron exporter ferroportin, thereby increasing macrophage iron content [52]. Variants in the *HFE* gene dysregulate this axis, leading to inappropriately low levels of hepcidin and macrophage-monocyte iron for the level of inflammation and/or systemic iron [53]. *Wildtype* HFE protein stably complexes with cellular TFR and to its cellular stabilizing factor, β_2_-microglobulin, decreasing uptake of iron-bound transferrin. These binding properties are believed to be crucial for macrophage-monocyte expression of *HFE* as well as for maintenance of iron-sensing mechanisms in the liver and gut, which are dysregulated by variants such as G845A [54, 55]. Reduced affinity of the G845A (C282Y amino-acid) variant of HFE for TFR and low hepcidin levels in G845A carriers result in reduced ability of macrophage-monocytes to retain iron [55]. In contrast, the C187G (H63D amino-acid) variant does not appear to influence HFE-TFR binding. Though the precise mechanisms of action of *HFE* are still not well understood, its role in regulating monocyte-macrophage iron content is well established; overall, *HFE* C282Y homozygosity results in systemic intracellular iron loading of cells *other than* macrophage-monocytes, due to a combination of unregulated gut iron absorption, decreased hepcidin synthesis in response to iron loading; unregulated gut iron absorption in individuals homozygous for this variant also leads to progressive iron deposition w(hemochromatosis) and oxidative stress in tissue parenchymal cells, and reduced macrophage-monocyte iron uptake [56]. Since the hepcidin-ferroportin axis evolved for the explicit purpose of iron-withholding and antimicrobial defense, it is therefore reasonable to anticipate that *HFE* variants result in blunted macrophage-mediated inflammatory responses. Published reports support the concept that low to moderate (*vs*. high) macrophage iron-loading has anti-inflammatory effects [57–60]. However, associations of sTFR levels and the sTFR-ferritin index with rapidity of onset of neuropathy in this study were not affected by adjusting for inflammation (hsCRP levels) in regression models, suggesting that iron availability may also impact susceptibility independent of inflammation or *HFE* genotype. Presence of *HFE* variants (and higher iron stores) in this study were also linked to lower inflammation in PWH, as reflected by hsCRP levels, likely owing to *HFE* linkage with other MHC Class I immunomodulatory genes and/or suppression of monocyte-macrophage cytokine production [58, 61]. Published data on the impact of *HFE* variants on cytokine-mediated inflammation is sparse and inconsistent; their effects may vary by cell type as well as the specific *HFE* variant [58, 62]. Ferroportin-mediated iron depletion of *Hfe*^-/-^ macrophages has been demonstrated to lead to decreased production of TNF-α and interleukin (IL-6) *in vitro* [58], while altered inflammatory cytokine levels have variably been reported with *HFE* variants and in iron deficiency [61–64]. Explanations for the association of higher systemic iron with reduced inflammation and slower onset of neuropathy in PWH also include: reduced replication of HIV in the iron-deficient macrophage-monocytes of people with *HFE* variants, and increased iron supply to neuronal mitochondria, which may somehow protect against cART-induced mitochondrial dysfunction [34]. Indeed, initiation of cART has been reported to increase sTFR levels, suggesting a state of functional iron deficiency in which cells with high metabolic demand for iron, such as neurons, may become iron-deficient; *HFE* variants could counteract these effects [65]. Whether higher systemic or cellular iron levels in individuals without *HFE* variants confer a similar benefit in delaying neuropathy onset and/or development of neuropathic pain during contemporary cART regimens remains to be determined in a larger study in which pain outcomes are also ascertained.

While biomarkers of inflammation other than hsCRP were not measured in this study, we used the sTFR-ferritin index, a well-accepted estimator of systemic iron stores, to adjust for the impact of inflammation due to HIV or other factors, since ferritin is a positive acute-phase protein and is often elevated in inflammatory states [66]. The sTFR is unaffected by infection and considerably less affected by inflammation than other iron biomarkers, such as ferritin [39]. The inverse correlation between time to onset of neuropathy and hsCRP levels suggests that pre-existing inflammation accelerates neurotoxicity, especially in genetically susceptible individuals. The association between *HFE* 187G and more delayed neuropathy onset supports this concept. Lack of a correlation between 845A alleles and time to neuropathy onset could be due to insufficient power, although differences in the cellular effects of these *HFE* variants have been suggested [67]. The lack of association between systemic iron stores and onset of neuropathy when self-reported non-whites were included in our analyses might be explained by the much lower frequency of *HFE* variants and/or higher inflammation in these individuals [40, 68]. The frequency of comorbidities or nutritional deficiencies, which are associated with inflammation independent of *HFE* genotype may also differ between these subpopulations, and we did not have information on these factors. Finally, since *HFE* gene variants influence both macrophage-mediated iron content and systemic iron levels, and we could not adjust more comprehensively for inflammation in our analyses, we cannot completely differentiate the effects of these variants on inflammation from their effects on iron status.

In summary, this represents the first study to directly evaluate the relationship between noninvasive measures of iron status and the time to onset of peripheral neuropathy after starting cART, shedding light on potential mechanisms underlying the previously reported association between *HFE* gene variants and reduced risk of HIV sensory neuropathy. Peripheral neuropathy and neuropathic pain remain important and treatment-limiting side effects for PWH who are treated with older drugs such as stavudine, as well as for PWH receiving protease inhibitors and other non-NRTI-based regimens [18, 20]; further studies should explore the influence of sTFR levels, estimated body iron stores, and *HFE* variants in these populations. Among PWH now aging into their 60s and beyond, neuropathy represents an important chronic comorbidity which can impair functional status and quality of life while increasing the risk of falls due to balance difficulties [69, 70]. Studies in larger cohorts are needed to overcome the sample size limitations encountered here and to evaluate potential interactions between iron levels, inflammation, cART, and *HFE* genotype in racially diverse HIV+ populations. Despite the availability of newer, less neurotoxic antiretroviral drugs, these findings still have important ramifications for HIV treatment in low-resource settings, where iron deficiency is endemic and neuropathic symptoms among PWH remain common.

## Supporting information

S1 Dataset De-identified dataset. (XLS)
